# Unconditional child support grant and outcomes on children: Experience from Thailand

**DOI:** 10.1371/journal.pone.0349423

**Published:** 2026-08-27

**Authors:** Kannika Damrongplasit, Kadir Atalay

**Affiliations:** 1 Faculty of Economics and Center of Excellence for Health Economics, Chulalongkorn University, Thailand; 2 School of Economics, University of Sydney, Australia; Thammasat University, THAILAND

## Abstract

**Background:**

Unconditional cash transfer emerges as a key instrument for reducing child poverty and promoting human capital development. However, limited evidence exists on the association with early childhood development outcomes in middle-income countries, particularly regarding unintended effects on household resource allocation and children’s technology exposure.

**Objective:**

This study examines the associations between Thailand’s unconditional Child Support Grant (CSG), early childhood development, access to educational materials, and electronic device usage.

**Methods:**

We analyze nationally representative data from the 2019 and 2022 Thailand Multiple Indicator Cluster Surveys (MICS). We employ propensity score matching with nearest neighbor matching to balance observed covariates between CSG recipients and non-recipients. Outcomes include child development, number of children’s books, children’s engagement with different types of toys, electronic device access, and daily screen time.

**Results:**

Children in households receiving the CSG demonstrate modest but statistically significant improvements in early childhood development. CSG receipt is associated with a 1.71 percentage point increase in developmental outcome, representing approximately 2% improvement relative to non-recipients. However, the analysis uncovers unexpected resource allocation patterns: recipient households possess significantly fewer children’s books, although children show increased engagement with homemade toys. In addition, CSG receipt is associated with greater access to electronic devices and longer screen time among children.

**Conclusion:**

Thailand’s unconditional cash transfer program successfully enhances child developmental outcomes. However, the findings also reveal complex household behavioral responses that suggest a need for complementary interventions, particularly to promote access to educational materials and provide guidance on appropriate electronic device use. The study offers important implications for policymakers seeking to maximize the developmental benefit of child-focused cash transfer programs in middle-income settings.

## Introduction

### Background

Investing in young children, particularly during the critical early childhood period of 0–5 years has shown to yield the highest returns compared to other age demographics. When children achieve their developmental potential during these formative years, it can have a profoundly beneficial effect on their current and future academic success, health indicators, employment opportunities, income, and various other behaviors [1–4]. From national perspective, such well-supported children are more likely to mature into competent adults who can contribute positively to the nation and society at large, for instance, through tax contributions, enhanced purchasing power, and the efficient production of goods and services that bolster the economy. This can also result in significant cost savings for the nation, such as decreased need for policy interventions later in life, lower healthcare costs, and reduced law enforcement expenses, among others.

Child support grant (CSG) has emerged as a crucial instrument for fostering human capital development and alleviating child poverty. By providing regular cash transfer to families with children, this grant aims to reduce financial barrier to essential services such as education, healthcare, and nutrition, thereby improving both immediate and long-term outcomes for children [5,6]. Many countries both developed and developing have implemented cash transfer program specifically aiming towards children [7–9]. There is substantial evidence that conditional CSG has positive impact in many dimensions of children such as poverty reduction [10], improved school enrolment and schooling outcomes [11], and leads to better health, nutritional and development outcomes [12–18]. Surprisingly, less empirical assessment is available for unconditional cash transfer and its impact on various child outcomes both in the context of high-income countries and LMICs. In this study, we try to fill this gap by providing empirical estimation on the effect of ‘unconditional’ CSG on early childhood development, the availability of child-specific goods such as books and toys, as well as children’s access and duration of electronic device usage by using nationally representative data from Thailand, a middle-income country.

Beyond traditional outcomes, this study examines the association between CSG and children’s access to electronic devices and screen time – outcomes that have received limited attention in the cash transfer literature. Electronic devices may support learning through access to educational applications and digital content. They may also enable caregivers to get information on contemporary parenting practices, potentially promoting children’s developmental outcomes. However, early and excessive exposure to electronic devices has been associated with adverse cognitive, language, and socio-emotional outcomes [19–21]. By increasing household disposable income, CSG may facilitate the acquisition of electronic devices, thereby influencing children’s access to digital technologies and engagement in screen-based activities. Understanding the relationship between CSG and children’s device access and duration of use is therefore important for identifying potential pathway through which the grant may affect child well-being.

Empirical findings of this research should be beneficial to both Thailand and other developing countries to learn from since the Thai CSG policy is an actual nationwide implementation of cash transfer unlike previous studies that tend to evaluate pilot stage or the implementation of such policy at a smaller scale like at village, community or city level rather than nationwide. Given limited budget, it is crucial for the government to evaluate how effective CSG has on children in various dimensions quantitatively. This can also assist policy makers in making effective policy intervention for children in the future.

### Unconditional child support grant

The Thai government introduced unconditional CSG starting from October 2015. The CSG in Thailand is non-contributory and non-conditional targeted cash transfer. According to UNICEF, the main objective of CSG is to provide financial support to eligible poor and near-poor family of young children so that essential needs can be met for the most vulnerable children; to promote access to services that can help children develop and able to reach their potential; and to break intergenerational poverty.

Thailand’s decision to implement unconditional CSG was motivated by the following reasons. First, the National 20-Year Strategy Plan identifies the development of future human capital as a strategic national priority, emphasizing the need to nurture and maximize the potential of children from birth across their lifespan, especially in the context of an aging society. Second, existing research shows that investing in children’s early years is a highly effective means of securing future productivity. Third, approximately one in five children experience multidimensional poverty. The lack of adequate opportunities and resources may hinder their ability to achieve their full potential, thereby adversely affecting both their future prospects and the country’s human capital and economic development. Fourth and finally, evidence from other countries demonstrates that child support grants are effective in alleviating child poverty and advancing long-term human capital objectives.

There are three main phases of unconditional CSG in Thailand. In the first phase that begins in October 2015, the government transfers cash worth 400 Baht a month to caregivers or mothers of Thai children aged zero to one year old. The income threshold to be eligible for CSG is 36,000 Baht per person per year. The second phase happens in March 2016 where the government expands the coverage of CSG to children under three years old and raises the grant to 600 Baht per month. The third phase takes place from March 2019 onward. The grant amount stays the same at 600 Baht per month; however, eligible children are now expanded to be below the age of six years old. The income threshold is also lifted to annual income of 100,000 Baht per person per year. According to UNICEF, the third expansion raises the number of children beneficiaries from 700,000 to 1.8 million.

To administer CSG, the CSG Operational Centre located within Department of Children and Youth is the main responsible entity with the support from many parties including Ministry of Finance for the transfer of payment through E-payment system, Ministry of Interior for targeting and registering the recipients, and Ministry of Public Health for implementation at local level. In addition, the Department of Children and Youth maintains an online platform that provides monthly monitoring data on the number of beneficiaries and CSG expenditures across provinces. National impact evaluations and assessments of the targeting mechanism was funded and implemented through a public-private partnership involving UNICEF, the Thailand Development Research Institute and the Thai Health Promotion Foundation [5].

Limited number of studies on the impact of unconditional CSG on different children outcomes have been conducted in high-income countries and LMICS, which return mixed findings. For child developmental outcomes, unconditional CSG program in Ecuador is found to have positive and significant impact on physical, cognitive and socio-emotional development for children aged less than 6 years old [22] whereas an unconditional cash transfer program in rural China has insignificant effect on children’s cognitive achievement for 7^th^-9^th^ graders [23]. Similarly, evidence from the United States suggest that unconditional cash transfer has no apparent effect on early childhood development in various dimensions including language, executive functioning, social-emotional problem as well as high-frequency brain activity during the first three years of children’s lives [24,25]. In term of health and nutritional outcomes, the South African CSG is found to significantly lower stunting in children aged less than 5 years old [26] while the CSG in Burkina Faso has neither effect on wasting nor stunting of children aged below 15 months old [27]. Focusing instead on healthcare access rather than health outcomes, a study from Vietnam finds that unconditional cash transfer has no significant effect on children’s outpatient or inpatient healthcare utilization or on out-of-pocket health expenditures [28]. Turning towards outcomes such as household’s investment on child-specific goods, we discover a few studies specifically assess these outcomes from Zambia [29] and the United States [30,31]. The Zambia’s study shows that unconditional CSG increases number of household with 3 or more books by 1.5 percentage point while empirical studies in the US demonstrate that spending on child-specific goods including books, toys, diapers and child related electronic devices has increased significantly among households that receive unconditional CSG.

Among existing studies on unconditional CSG, the evaluation of Thai CSG by UNICEF is the most closely related to our research [5]. The main contributions of our study are as follows. We use more updated data to perform the analysis. In particular, we use both 2019 and 2022 Multiple Indicator Cluster Survey data (MICS) as opposed to previous research that uses data from 2016–2017. This allows us to assess the impact of the third phase of CSG implementation in Thailand and see its longer term effect on children while previous study by UNICEF identifies impact of the second phase only. Furthermore, the data employed in the UNICEF’s study focuses on 9 provinces while MICS data in this study is a nationally representative data covering the entire nation. This enables us to assess the association between CSG receipt and a range of child outcomes across all regions of Thailand. Additionally, this study considers different set of outcome variables. We focus our attention on overall early childhood development, availability of child-specific items such as books and toys, and children’s access and duration of electronic device usage while previous study looks at child nutritional outcome, access to post-natal service and the role of CSG on women empowerment.

## Data and method

### Sample and variables

We use 2019 and 2022 Thailand Multiple Indicator Cluster Survey (MICS). The MICS survey has been collected jointly by UNICEF and the National Statistical Office (NSO) of Thailand. Permission to use MICS data has been granted by the National Statistical Office of Thailand under contract number 66/2565 and 5/2567. Approval to conduct this study has been granted by the Research Ethics Review Committee for Research Involving Human Subjects: The Second Allied Academic Group in Social Sciences, Humanities and Fine and Applied Arts at Chulalongkorn University (Certificate of Research Approval No. 453/66). The MICS data were first access for research purposes on 15/01/2024. The data are de-identified, third-party data and informed consent for secondary data analysis is not required. They are repeated cross-sectional data of nationally representative households in Thailand. The dataset covers all five main regions of Thailand as well as urban and rural areas. There are five main components to the survey including questionnaire for household, individual women, individual men, children under 5 and children aged 5–14 years old. Our research focuses on two components namely household and children under 5 questionnaires. This is because by design children aged below 6 years old are eligible to get unconditional Thai CSG. The household component collects information on all the household members who reside within the household while the child under 5 years old component is answered by a mother or a caretaker who lives in the household. By merging the two components together and combining the two years of data, we can obtain necessary information whether a household receives any CSG; basic characteristics of household, head of household and children; different dimensions of child related outcomes. With two waves of data, we can assess the impact of CSG policy over the longer-term. Since this study’s outcomes are children related outcomes, the final unit of analysis is child-level.

Our sample consists of 11464 observations of which 6477 children (56.5%) reside in the households that receive unconditional CSG while 4987 children (43.5%) live in the non-CSG recipient households. There are altogether 6819 observations in 2022 and 4645 observations in 2019. The treatment variable comes from a question in the MICS’s household questionnaire of whether a household or anyone within a household has received unconditional CSG. The outcome variables consist of (i) an indicator variable of whether a child is developmentally on track, (ii) number of children’s books that a child has, (iii) whether a child plays with homemade toys, (iv) whether a child plays with toys from shops or commercial toys, (v) whether a child plays with household objects, (vi) whether a child plays with electronic devices such as mobile phone, tablet, game console, and (vii) the number of hours a child plays electronic devices in a day. For a variable capturing overall child development outcome, we follow the definition used by UNICEF, which has changed over our sample periods. In 2019, children aged 36–59 months are regarded as being developmentally on track if they exhibit proficiency in at least three of the four following domains: literacy-numeracy, physical, social-emotional, and cognitive development. For each domain, there are list of questions, where a caregiver indicates whether a child can achieve a minimum of 50%. In 2022, there are 20 closed-response inquiries where a caretaker rates whether a child can perform. Each child is allocated a computed score out of 20 points. A child is deemed to be developmentally on track if his/her score surpasses a particular benchmark, which escalates with the child’s age. We then create a binary variable equals to 1 to capture a child being developmentally on track and 0 otherwise. Our sample consists of children aged between 3–5 years old as the outcome variables we consider are only available for children in this age group. The list of questions used in MICS to assess early childhood development in 2019 and 2022 is provided in S1 Table.

### Propensity Score Matching

The main objective of this research is to assess the impact of receiving unconditional CSG on various children outcomes. With repeated cross-sectional data, we employ propensity score matching method to estimate such impact. Since participation in the CSG is non-random, direct comparisons between recipients and non-recipients may yield biased estimates due to underlying socioeconomic differences. Propensity score matching addresses this issue by creating a comparison group that closely resembles CSG recipients based on observed characteristics. By matching children with similar propensity scores, the method reduces selection bias arising from observable covariates and improves the comparability of the treatment and control groups. Propensity score matching has been used extensively in health economics and labor economics related literature [32–37]. With this approach, we first estimate propensity score, $p\left(x_{i}\right)=p(d_{i}=\text{1}|x_{i})$, which is a conditional probability of being assigned into the treatment group. In this case, the treatment group (i.e., $d_{i}=\text{1}$) captures those children whose families receive unconditional CSG and the control group (i.e., $d_{i}=\text{0}$) are those children whose families do not receive unconditional CSG. For $x_{i}$ which are the variables used for predicting propensity score, they consist of socioeconomic demographic factors of household, household head, caretaker as well as children and are listed at the bottom panel of Table 1. Binary logistic regression is used for estimating the propensity score in this research. Under the following assumptions of overlapping propensity score between treatment and control groups

**Table 1:** Summary statistics.

	2019 and 2022			2022			2019		
	Non Receipt	Receipt	All Sample	Non Receipt	Receipt	All sample	Non Receipt	Receipt	All sample
***Treatment Variable***									
*CSG Receipt (binary)*	0	1	0.565	0	1	0.762	0	1	0.275
# of obs (%)	4987 (43.5%)	6477 (56.5%)	11464 (100%)	1621 (23.8%)	5198 (76.2%)	6819 (100%)	3366 (72.5%)	1279 (27.5%)	4645 (100%)
***Outcome Variables***									
*Child Development (binary)*	0.876	0.892	0.885	0.906	0.905	0.905	0.862	0.843	0.856
# of obs (%)	4987 (43.5%)	6477 (56.5%)	11464 (100%)	1621 (23.8%)	5198 (76.2%)	6819 (100%)	3366 (72.5%)	1279 (27.5%)	4645 (100%)
*Children Book (# of books)*	3.365	2.516	2.886	3.912	2.537	2.864	3.102	2.433	2.918
# of obs (%)	4983 (43.5%)	6474 (56.5%)	11457 (100%)	1619 (23.8%)	5197 (76.2%)	6816 (100%)	3364 (72.5%)	1277 (27.5%)	4641 (100%)
*Play Homemadetoy (binary)*	0.638	0.673	0.658	0.698	0.698	0.698	0.610	0.574	0.600
# of obs (%)	4978 (43.5%)	6470 (56.5%)	11448 (100%)	1620 (23.8%)	5194 (76.2%)	6814 (100%)	3358 (72.5%)	1276 (27.5%)	4634 (100%)
*Play Toyshop (binary)*	0.982	0.983	0.982	0.992	0.983	0.985	0.977	0.981	0.978
# of obs (%)	4984 (43.5%)	6476 (56.5%)	11460 (100%)	1619 (23.8%)	5197 (76.2%)	6816 (100%)	3365 (72.5%)	1279 (27.5%)	4644 (100%)
*Play Householdobject (binary)*	0.878	0.881	0.879	0.866	0.880	0.877	0.883	0.883	0.883
# of obs (%)	4982 (43.5%)	6470 (56.5%)	11452 (100%)	1621 (23.8%)	5193 (76.2%)	6814 (100%)	3361 (72.5%)	1277 (27.5%)	4638 (100%)
*Play Electronic (binary)*	0.735	0.793	0.767	0.801	0.819	0.815	0.703	0.684	0.698
# of obs (%)	4982 (43.5%)	6471 (56.5%)	11453 (100%)	1621 (23.8%)	5194 (76.2%)	6815 (100%)	3361 (72.5%)	1277 (27.5%)	4638 (100%)
*Duration Electronic (hours)*	1.066	1.202	1.145	1.361	1.272	1.293	0.902	0.856	0.890
# of obs (%)	3625 (41.5%)	5105 (58.5%)	8730 (100%)	1294 (23.4%)	4242 (76.6%)	5536 (100%)	2331 (73%)	863 (27%)	3194 (100%)
***Control Variables***									
*Child Age (years)*	3.850	3.556	3.684	3.569	3.494	3.512	3.985	3.811	3.937
*Child Gender (binary)*	0.518	0.514	0.515	0.531	0.514	0.518	0.511	0.513	0.512
*HeadHH Gender (binary)*	0.588	0.582	0.585	0.606	0.579	0.585	0.579	0.595	0.584
*HeadHH Age (years)*	51.260	51.054	51.144	50.825	51.186	51.101	51.470	50.514	51.207
*HH Number (# of people)*	4.753	5.110	4.955	4.742	5.090	5.007	4.758	5.191	4.877
*Caretaker Primary (binary)*	0.299	0.260	0.277	0.248	0.255	0.253	0.324	0.282	0.312
*Caretaker Secondary (binary)*	0.318	0.434	0.384	0.258	0.433	0.392	0.347	0.438	0.372
*Caretaker Vocational (binary)*	0.100	0.114	0.108	0.085	0.112	0.105	0.107	0.122	0.111
*Caretaker University (binary)*	0.218	0.159	0.185	0.327	0.167	0.205	0.165	0.127	0.155
*Caretaker UniPlus (binary)*	0.035	0.007	0.019	0.047	0.008	0.017	0.029	0.005	0.023
*2nd Wealth Decile (binary)*	0.094	0.108	0.102	0.057	0.104	0.093	0.112	0.126	0.116
*3rd Wealth Decile (binary)*	0.088	0.125	0.109	0.062	0.122	0.108	0.101	0.137	0.111
*4th Wealth Decile (binary)*	0.108	0.115	0.112	0.085	0.113	0.106	0.120	0.125	0.121
*5th Wealth Decile (binary)*	0.100	0.119	0.111	0.096	0.117	0.112	0.102	0.126	0.108
*6th Wealth Decile (binary)*	0.109	0.118	0.114	0.110	0.120	0.118	0.109	0.109	0.109
*7th Wealth Decile (binary)*	0.104	0.104	0.104	0.112	0.107	0.109	0.100	0.088	0.097
*8th Wealth Decile (binary)*	0.107	0.085	0.095	0.130	0.090	0.099	0.097	0.066	0.088
*9th Wealth Decile (binary)*	0.100	0.062	0.079	0.131	0.061	0.078	0.085	0.065	0.079
*10th Wealth Decile (binary)*	0.100	0.044	0.068	0.153	0.046	0.071	0.074	0.038	0.064
*Central (binary)*	0.220	0.136	0.172	0.143	0.106	0.115	0.258	0.255	0.257
*North (binary)*	0.165	0.172	0.169	0.173	0.181	0.179	0.162	0.134	0.154
*Northeast (binary)*	0.288	0.333	0.313	0.232	0.349	0.321	0.314	0.265	0.301
*South (binary)*	0.239	0.335	0.294	0.308	0.340	0.332	0.206	0.317	0.237
*Urban (binary)*	0.431	0.387	0.406	0.543	0.408	0.440	0.377	0.301	0.356
# of obs (%)	4987 (43.5%)	6477 (56.5%)	11464 (100%)	1621 (23.8%)	5198 (76.2%)	6819 (100%)	3366 (72.5%)	1279 (27.5%)	4645 (100%)


$$\begin{array}{c} \text{0}<\text{p}\left(x_{i}\right)<\text{1} \end{array}$$ (1)

and ignorable treatment selection once controlling for observable factors,


$$\begin{array}{c} y_{\text{1}i},y_{\text{0}i}⊥\;d_{i}\;|x_{i}, \end{array}$$ (2)

the following properties would hold:


$$\begin{array}{c} y_{\text{1}i},y_{\text{0}i}⊥\;d_{i}\text{\textasciitilde{}}|\text{\textasciitilde{}}p(x_{i}) \end{array}$$ (3)

and


$$\begin{array}{c} x_{i}⊥d_{i}\text{\textasciitilde{}}|\;p(x_{i}) \end{array}$$ (4)

Equation (3) implies that once conditioning for propensity score the assignment to treatment is independent of outcomes in both treated and untreated states (y_1_ and y_0_) while equation (4) indicates that the distribution of covariates $x_{i}$ is the same across treatment and control groups for those with similar propensity score. In this study, one to one nearest neighbor’s propensity score matching with replacement is used to match treatment and control observations with the closest predicted propensity score. Once the matched treated and controlled observations are made, average treatment effect (ATE) which is the average of outcome difference across all matched pairs


$$\begin{array}{c} ATE=\frac{\text{1}}{n}\Sigma \left(y_{i}-y_{j}\right) \end{array}$$

and average treatment on the treated (ATET) which is the average of outcome difference across the matched pairs only for the treated observations


$$\begin{array}{c} ATET=\frac{\text{1}}{n_{T}}\Sigma \left(y_{i}-y_{j}\right) \end{array}$$

can be computed to give the estimates of receiving unconditional CSG on different children outcomes. Here, y_i_ is the outcome of the treated unit, y_j_ is the outcome of the matched control unit, n is total number of treated and matched control units, and n_T_ is number of treated units only. ATE measures the expected impact of CSG receipt for a randomly selected child in the population and is particularly relevant for assessing the potential effects of expanding the program to a broader population of children. In contrast, the ATET measures the average effect of CSG receipt among current beneficiaries and is relevant for evaluating the program’s effectiveness for existing recipients.

## Results

### Descriptive statistics

Summary statistics from Table 1 demonstrates that for overall sample of children who reside in households that receive CSG have on average 1.6 percentage point higher chance of being developmentally on track comparing to those in the CSG non-recipient households ((0.892–0.876)*100). There is also higher tendency for children whose families receive CSG to play more homemade toy, have better access to electronic devices, as well as spend more time on using these electronic devices. When comparing overtime between 2019 and 2022 regardless of grant receiving status, there appears to be an overall improvement in child development, less number of children books available, more homemade and manufactured toys available to children, and on average about 11.7 percentage point ((0.815–0.698)*100) higher chance for children to use electronic devices with an average of 0.136 hours (1.293–0.89) longer of use in 2022 relative to 2019. For control variables which is found in the bottom panel of Table 1, the average age of the children in our sample appears to be slightly younger in 2022 at 3.5 years old comparing to 3.9 years old in 2019. The gender of the children is evenly split between boys and girls in both periods. Characteristics of household and caretakers appear to be similar across the two years with about 58% of household head being male, has an average age of 51 years old, and the number of household members are at around 5 persons per household. Caretaker’s education appears to increase slightly with larger fraction that graduates with university degree or higher. To control for regional differences, we include indicator variables for five geographical regions of Thailand: Central (excluding Bangkok), North, Northeast, South, and East. Bangkok serves as the reference category in our model. With the combined sample of two periods, about 41% of the observations reside in urban area. Finally, to capture household’s economic status, ten wealth deciles are included as control variables with the first wealth decile serves as a reference category. The wealth index is a composite measure of household economic status. It is derived using principal components analysis (PCA) and incorporates information on ownership of consumer goods, housing characteristics, access to water and sanitation facilities, and other attributes associated with household wealth. Households are subsequently ranked and divided into wealth deciles. A detailed description of the construction of this index is provided by NSO [38].

### Overlapping propensity score and covariates balancing

Table 2 provides regression result of the estimated propensity score using binary logit model. Variables that are positively and statistically significant in predicting whether families receive unconditional CSG are number of household members, caretaker having secondary or vocational education, region of residence not in Bangkok and living in urban area. On the other hands, factors that have significant and negative effect on being CSG recipients comprise child age, age of head of household, caretaker having more than bachelor degree, and higher household wealth as measured by 2^nd^ to 10^th^ wealth deciles as compared to the 1^st^ wealth decile. The effect of wealth appears to be monotonically negatively increasing with higher wealth decile. The signs of estimated coefficients match with our prior expectation as the Thai unconditional CSG aims to provide financial support to eligible poor and near-poor families that tend to have lower socioeconomic status.

**Table 2:** Propensity score estimation using binary logistic model.

	*CSG receipt*
*Child Age*	−0.471***
	(0.0255)
*Child Gender*	−0.0379
	(0.0400)
*HeadHH Gender*	−0.0216
	(0.0407)
*HeadHH Age*	−0.00437***
	(0.00153)
*HH Number*	0.126***
	(0.0127)
*Caretaker Primary*	0.0935
	(0.125)
*Caretaker Secondary*	0.544***
	(0.125)
*Caretaker Vocational*	0.536***
	(0.136)
*Caretaker University*	0.158
	(0.133)
*Caretaker UniPlus*	−0.835***
	(0.215)
*2nd Wealth Decile*	−0.186**
	(0.0881)
*3rd Wealth Decile*	0.000649
	(0.0880)
*4th Wealth Decile*	−0.316***
	(0.0867)
*5th Wealth Decile*	−0.207**
	(0.0883)
*6th Wealth Decile*	−0.341***
	(0.0882)
*7th Wealth Decile*	−0.429***
	(0.0908)
*8th Wealth Decile*	−0.561***
	(0.0938)
*9th Wealth Decile*	−0.745***
	(0.102)
*10th Wealth Decile*	−0.851***
	(0.113)
*Central*	0.728***
	(0.110)
*North*	1.311***
	(0.112)
*Northeast*	1.309***
	(0.107)
*South*	1.501***
	(0.107)
*Urban*	0.0908**
	(0.0432)
_cons	0.441**
	(0.206)
N	11464

Note: Standard errors in brackets, * p < 0.10, ** p < 0.05, *** p < 0.01

**Fig 1:**
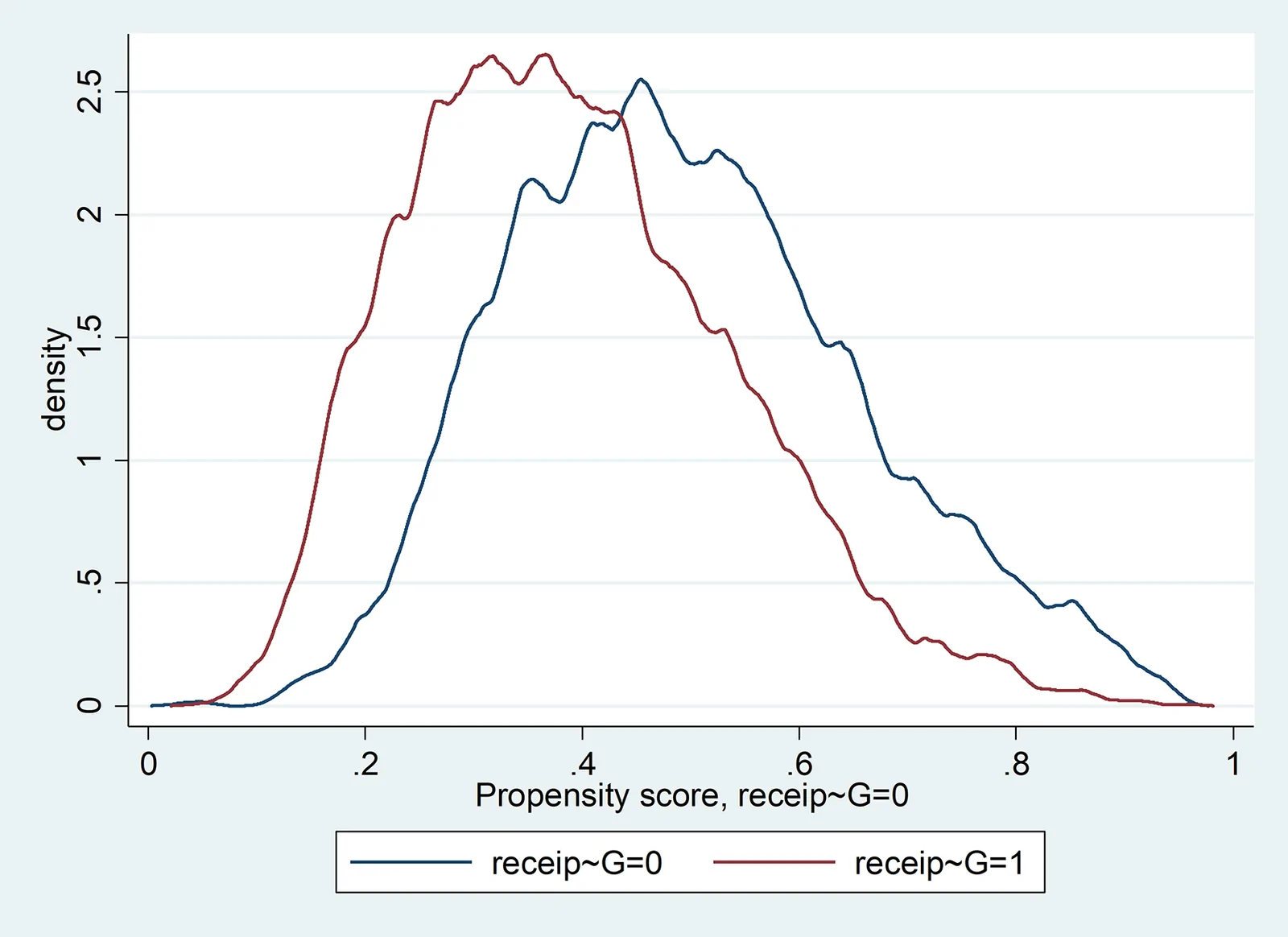
Density of estimated propensity score of the treatment and control group.

**Fig 2:**
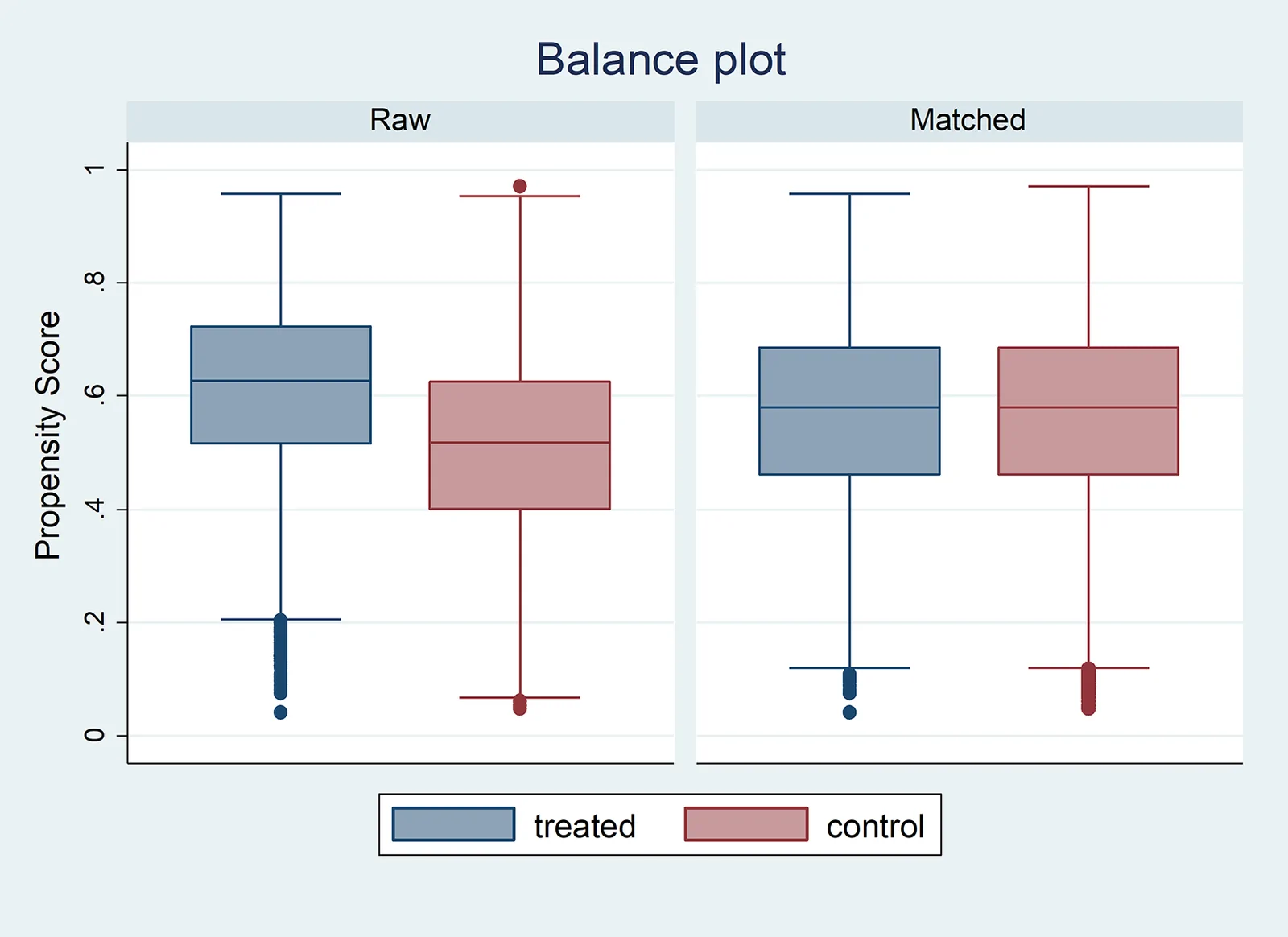
Balance plot of estimated propensity score of the raw and matched treatment and control group.

To check overlapping of propensity score between the treatment and control groups, Fig 1 shows density of estimated propensity score for the CSG recipients and the non-CSG recipients. There seems to be substantial overlap between the two groups across the 0.05 to 0.95 range of propensity scores, indicating a strong common support region. Fig 2 also presents box plots of estimated propensity score for the matched treatment and control groups. It is clear from the box plots that the 25^th^ percentile, the median and the 75^th^ percentile of the estimated propensity score are very similar for the treatment and the matched control observations. This strong overlap of estimated propensity score implies that we can compare the treatment and matched control groups more reliably, since most treated individuals have comparable counterparts in the control group. This strong overlap supports the validity of using propensity score methods for estimating causal effects in this study.

Table 3 reports the standardized mean differences and variance ratios for covariates used in the propensity score estimation, both before and after the matched is made. The results show that, after matching, the standardized differences for all covariates are close to zero, most are well below the commonly accepted threshold of 0.1, indicating excellent covariate balance between the treatment and matched control groups. This implies that the means of the covariates are nearly identical across the two groups, suggesting that the matching procedure has successfully reduced systematic differences in observed characteristics. Additionally, the variance ratios after matching are generally close to one, further confirming that the distributions of covariates are similar in both groups.

**Table 3:** Covariate balancing (Mean difference between treatment and control group).

	Standardized differences		Variance ratio	
	Raw	Matched	Raw	Matched
Child Age	-.3715926	.0031438	1.326669	1.171393
Child Gender	-.0080758	.0071569	1.000456	.9995016
HeadHH Gender	-.0123179	-.0132718	1.004218	1.004522
HeadHH Age	-.0144942	.0011818	1.073563	1.040077
HH Number	.2023312	-.0313064	1.259609	.893056
Caretaker Primary	-.0869393	.0333898	.9179338	1.034133
Caretaker Seconda	.242199	-.0141612	1.133111	.9933329
Caretaker Vocatio	.0455826	-.0204123	1.123116	.9501351
Caretaker Univers	-.1502791	.0024784	.785768	1.00406
Caretaker UniPlus	-.1942372	.0037566	.2127434	1.026238
2nd Wealth Decile	.0465575	-.0267635	1.131338	.9321569
3rd Wealth Decile	.1199845	.0456924	1.361541	1.124553
4th Wealth Decile	.0218868	-.0069371	1.055405	.9829838
5th Wealth Decile	.0609724	-.0127317	1.165281	.9691194
6th Wealth Decile	.0266833	.0345741	1.067075	1.087627
7th Wealth Decile	-.0015512	-.0114352	.9959326	.9707419
8th Wealth Decile	-.0760197	.0120092	.8113379	1.034218
9th Wealth Decile	-.1388728	-.0092356	.6475962	.9721802
10th Wealth Decil	-.215363	-.0097632	.4719334	.9667678
Central	-.2231075	-.0307095	.6820119	.9481317
North	.016836	-.0119699	1.030249	.9786825
Northeast	.0974306	.0387571	1.083422	1.031767
South	.2131358	.005718	1.22393	1.005046
Urban	-.0910522	-.0271977	.9667566	.9894333

**Fig 3:**
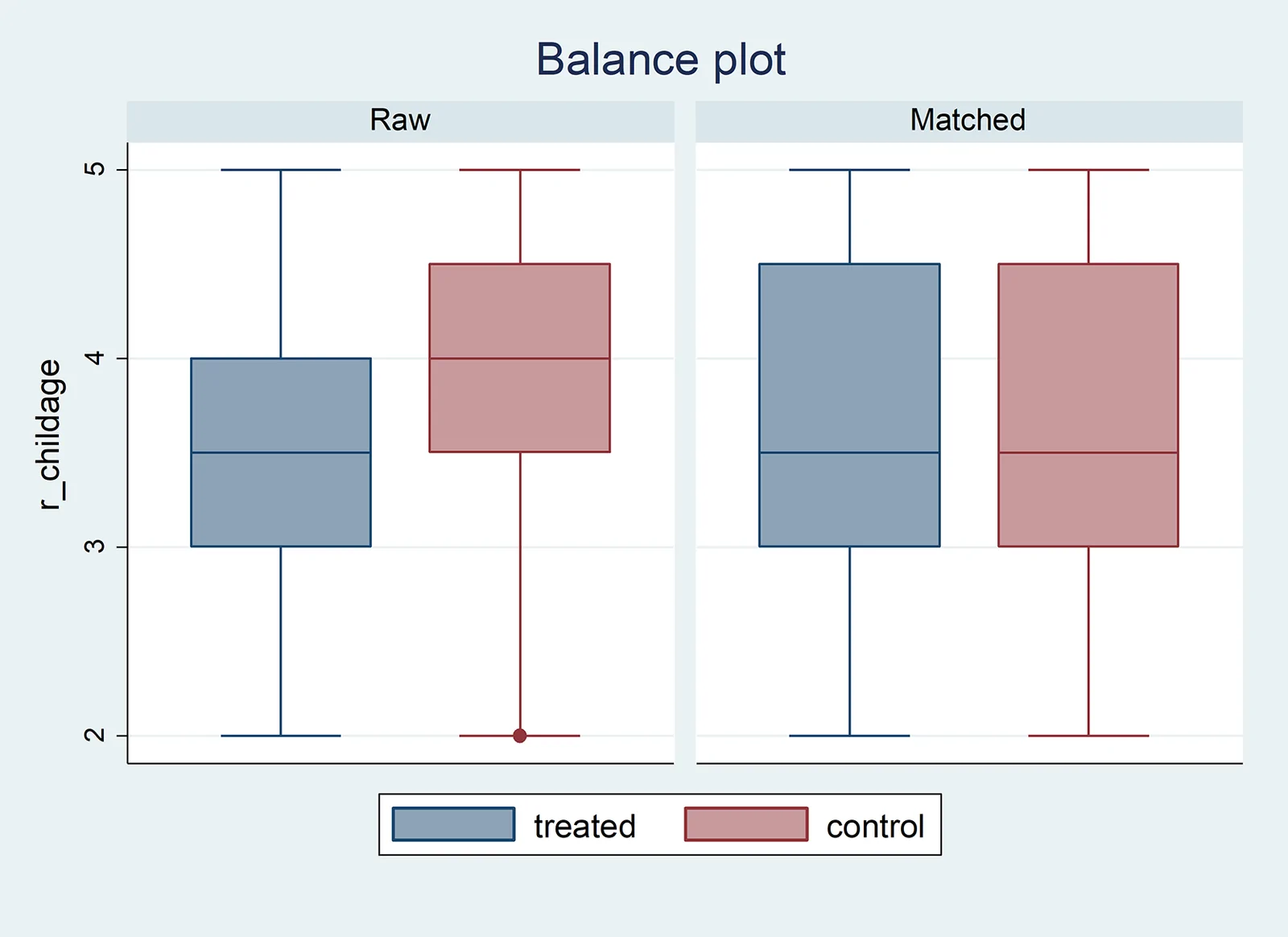
Covariates balancing for continuous variables – child age.

**Fig 4:**
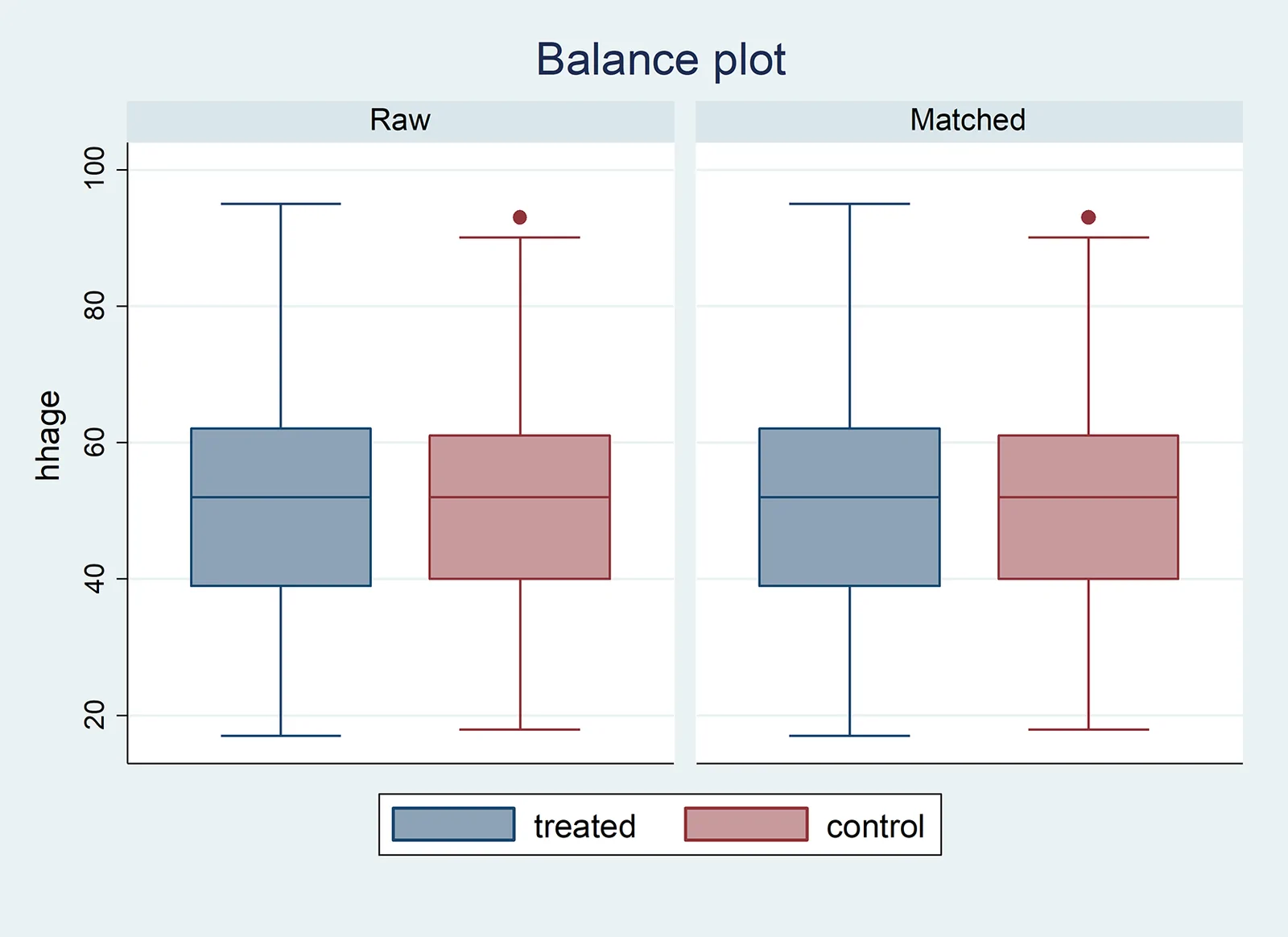
Covariates balancing for continuous variables – head of household age.

**Fig 5:**
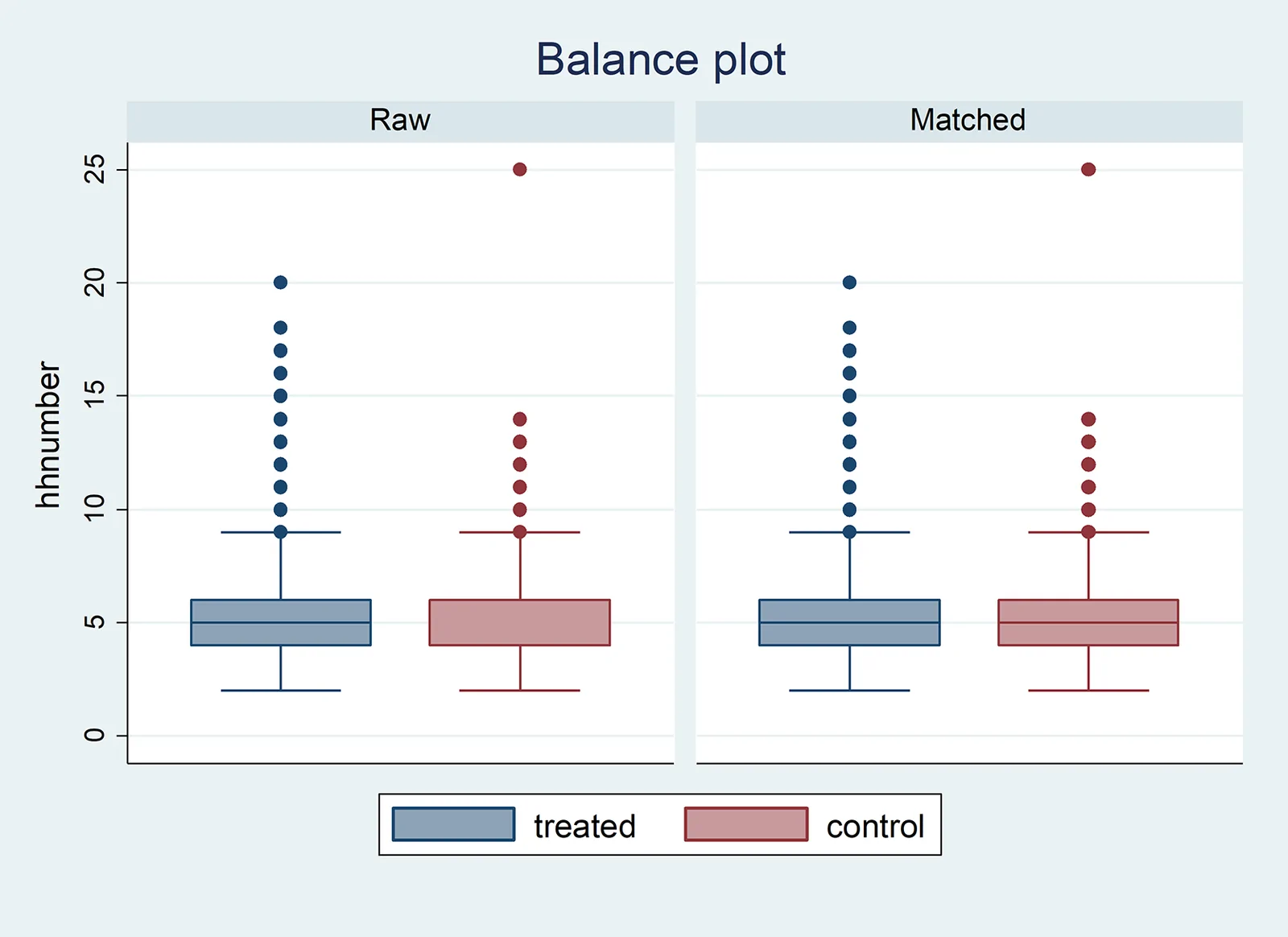
Covariates balancing for continuous variables – household number.

For continuous variables like child age, head of household age, and number of family members, Figs 3 and 4 also provides box plots for the 25^th^ percentile, the median, and the 75^th^ percentile of these variables for the treatment and matched control counterparts for each respective variables. Once again, the box plots appear to be resemblance for the matched observations giving additional evidence of covariate balancing among continuous variables Fig 5.

### Estimated results

Table 4 gives estimated ATE and ATET for both 2019 and 2022 combined MICS data as well as for each year separately. The combined analysis across 2019 and 2022 shows statistically significant, though modest, positive association between receiving the unconditional CSG and overall early childhood development. Specifically, both the average treatment effect (ATE = 0.0171) and average treatment effect on the treated (ATET = 0.0188) for the Child Development outcome are positive and statistically significant at the 5% level, suggesting a modest but meaningful improvement in development for children who receive the grant. In practical terms, receiving unconditional CSG leads to 1.71 percentage point increase in overall early childhood development for any randomly selected child and approximately 1.88 percentage point increase for children whose families actually receive unconditional CSG. Comparing to the average of non-CSG recipients, this result represents approximately 2% increase in early childhood development once CSG is distributed ((0.0171/0.876)*100). The statistically significant result found in the pooled analysis points to a potential long-term benefit of grant receipt on child development over time.

**Table 4:** Estimated ATE and ATET.

Outcome	2019 and 2022		2022		2019	
	ATE	ATET	ATE	ATET	ATE	ATET
*Child Development*	0.0171**	0.0188**	0.0186	0.0206	−0.0104	−0.016
	(0.002, 0.032)	(0.001, 0.037)	(−0.006, 0.043)	(−0.008, 0.049)	(−0.037, 0.017)	(−0.05, 0.018)
# of obs	11464		6819		4645	
*Children Book*	−0.1877***	−0.1567**	−0.3902***	−0.4466***	−0.2606**	−0.2502*
	(−0.317, −0.058)	(−0.305, −0.008)	(−0.58, −0.2)	(−0.648, −0.245)	(−0.515, −0.006)	(−0.504, 0.003)
# of obs	11457		6816		4641	
*Play Homemadetoy*	0.0239**	0.0094	−0.0144	−0.0265	−0.0297	−0.027
	(0.002, 0.046)	(−0.016, 0.035)	(−0.048, 0.019)	(−0.064, 0.011)	(−0.069, 0.01)	(−0.071, 0.017)
# of obs	11448		6814		4634	
*Play Toyshop*	0.0039	0.0029	−0.0087**	−0.0089**	0.0114**	0.0102
	(−0.002, 0.01)	(−0.004, 0.01)	(−0.016, −0.001)	(−0.017, −0.0003)	(0.002, 0.021)	(−0.005, 0.026)
# of obs	11460		6816		4644	
*Play Householdobject*	−0.0012	0.0019	0.0047	0.0109	−0.0204	−0.0051
	(−0.017, 0.015)	(−0.017, 0.02)	(−0.027, 0.036)	(−0.027, 0.049)	(−0.051, 0.011)	(−0.033, 0.023)
# of obs	11452		6814		4638	
*Play Electronic*	0.0833***	0.0876***	0.026	0.0284	−0.0128	0.0008
	(0.063, 0.104)	(0.062, 0.113)	(−0.005, 0.057)	(−0.007, 0.064)	(−0.05, 0.024)	(−0.041, 0.042)
# of obs	11453		6815		4638	
*Duration Electronic*	0.1389***	0.1094*	−0.168**	−0.201**	0.0241	−0.0064
	(0.056, 0.222)	(−0.005, 0.224)	(−0.319, −0.017)	(−0.382, −0.02)	(−0.085, 0.133)	(−0.125, 0.112)
# of obs	8730		5536		3194	

Note: In parentheses are 95% confidence intervals.

To our surprise, the impact of receiving unconditional CSG on number of children books in the household (Children Book) is consistently negative and statistically significant with estimated ATE equals to −0.19 and estimated ATET equals to −0.16 while both being statistically significant at 5% level. These results indicate that CSG recipients have fewer books than non-grant recipients.

The estimated effects of the unconditional CSG on children’s engagement with different types of toys notably homemade toys*,* toys from toy shops*,* and household object are mixed and vary by year. Our pooled analysis shows that receiving the grant is associated with a small but statistically significant increase in the likelihood of playing with homemade toys, with ATE of 0.024. This implies a 2.4 percentage point increase in the likelihood that any randomly selected child receiving unconditional CSG would play with homemade toys. However, the estimated ATET for this outcome is not statistically significant, suggesting that the effect is not as strong among those who actually received the grant. The effects for playing toys from toy shops (Play Toyshop) and playing household objects (Play Householdobject) are statistically insignificant.

Lastly, the unconditional CSG has strong association with children’s engagement with electronic devices, measured through both the likelihood of playing with such devices (Play Electronic) as well as the amount of time spent doing so (Duration Electronic). Being grant recipient is associated with a significant increase in both children’s use of electronic devices as well as the duration of use. In the overall analysis, the ATE for Play Electronic is 0.0833 and the ATET is 0.0876, both statistically significant at the 1% level, suggesting that for any randomly selected child receiving unconditional CSG has on average 8.3 percentage point higher chance of playing with electronic devices such as computers, tablets, mobile phones, or gaming consoles. Among children who actually receive CSG, this effect is even greater at 8.8 percentage point increase. Additionally, Duration Electronic shows a significantly longer usage time, with an ATE of 0.139 and an ATET of 0.109, indicating approximately 0.139 and 0.109 longer hours of screen time spent per day for any random children receiving CSG and for children whose families actually receive CSG, respectively.

### Sensitivity analyses

We conduct sensitivity analyses using binary logit model for binary outcomes and ordinary least square (OLS) regression for all outcomes, adjusting for the same covariates. Table 5 reports both average marginal effects (AMEs) from binary logit model and the estimated OLS coefficient associated with *CSG receipt* for each outcome. The full estimation results are presented in S2 and S3 Tables.

**Table 5:** Sensitivity analyses from binary logit model and ordinary least square estimation.

Outcome	Average Marginal Effect of CSG Receipt Binary Logit Model	Estimated Coeffcient of CSG Receipt Ordinary Least Square Estimation
*Child Development (binary)*	0.0170***	0.0174***
	(0.00622)	(0.00631)
*Children Book (# of books)*	N/A	−0.175***
		(0.0547)
*Play Homemadetoy (binary)*	0.0241**	0.0243***
	(0.00937)	(0.00941)
*Play Toyshop (binary)*	0.00257	0.00279
	(0.00267)	(0.00262)
*Play Householdobject (binary)*	−0.00742	−0.00713
	(0.00647)	(0.00645)
*Play Electronic (binary)*	0.0806***	0.0805***
	(0.00807)	(0.00817)
*Duration Electronic (hours)*	N/A	0.202***
		(0.0284)

Overall, both alternative estimation approaches yield results that are highly consistent with the propensity score matching (PSM) estimates. For child development, our estimated ATE from PSM is 0.0171, while the corresponding AME from the binary logit model is 0.017 and the OLS coefficient is 0.0174. All three estimates are statistically significant, indicating that the findings are robust across estimation methods. For the number of books, the OLS coefficient associated with CSG receipt is −0.175 and is statistically significant, closely matching the PSM estimates of −0.187. Similar consistency is observed for *Play Homemadetoy*, where the estimated effects are statistically significant across all three models, with estimates of 0.0239, 0.0241, and 0.02143 from the PSM, binary logit, and OLS models, respectively. In contrast, all three models indicate no statistically significant effect of CSG receipt on *Play Toyshop* and *Play Householdobject*. For electronic device use, the estimated effects from PSM, binary logit, and OLS models are all approximately 0.08, suggesting a high degree of consistency across estimation approaches. Finally, for *Duration Electronic*, the PSM estimate is statistically significant at 0.14, while the OLS coefficient is also statistically significant and slightly higher in magnitude (0.202).

Taken together, the sensitivity analyses demonstrate that our findings are robust to alternative estimation strategies. The consistency of the estimated effects across propensity score matching, binary logit, and OLS models increases confidence that the main results are not driven by the choice of estimation method and suggests that the identifying assumptions underlying these approaches are reasonably satisfied.

## Discussion

This study uses 2019 and 2022 MICS data that cover all regions of Thailand and attempts to assess the association between receiving unconditional CSG and various children outcomes including overall early childhood development; the availability of child-specific goods as measured by number of children books and different kinds of toys that children play; children’s access to electronic devices and duration of electronic devices’ usage. Propensity score matching method is used for analysing the data. Our findings that unconditional CSG has positive and significant association with overall child development are consistent with evidence from Ecuador, a country with the same World Bank’s income classification as Thailand. Ecuador’s unconditional CSG is found to have positive and significant effect on physical, cognitive and socio-emotional development for children aged less than 6 years old [22]. However, our results differ from the experience of a high income country like the United States where unconditional cash transfer has insignificant effect on language, executive functioning or socio-emotional development of the children [24,25]. There is one previous study done by UNICEF that evaluates the same Thai CSG program, however, during the second phase of its implementation and by using data from nine provinces [5]. In contrast, our study examines the third phase of the CSG program and by using nationally representative data. The UNICEF’s study focuses on a specific dimension of child development—child nutritional status, measured by weight-for-height (wasting)—and finds that the CSG reduces the prevalence of wasting among Thai children by approximately four percentage points [5]. This improvement in children’s health-related outcomes is consistent with our finding that unconditional CSG receipt is positively and significantly associated with overall early childhood development, increasing the probability of being developmentally on track by approximately 1.7 percentage point. In terms of population-level implications, approximately 1.8 million children in Thailand were receiving the Child Support Grant (CSG) in 2024. Applying the estimated 1.7 percentage point increase in the likelihood of being developmentally on track among grant recipients suggests that approximately 30,600 additional children (0.017 × 1.8 million) may achieve age-appropriate developmental milestones. These estimates underscore the potential public health significance of the CSG in promoting early childhood development. Furthermore, both UNICEF’s study and this research’s finding together imply that the beneficial effect of unconditional CSG in Thailand appears to be persistent over a long period of time.

One of the most unexpected findings is the significant decline in the number of children books following unconditional CSG receipt. Specifically, our results suggest that, on average, any randomly selected households receiving CSG have approximately 0.19 fewer children’s books compared to those non-grant recipient counterparts. To contextualize this magnitude, the mean number of children books in non-recipient households in our sample is 3.365 books. A reduction of 0.19 books following grant receipt therefore represents approximately 5.65% decline relative to the comparison group mean. Given that many households in our sample have very few children books to begin with, even small reductions may meaningfully constraint opportunity for children to develop reading and language skills. This contrasts with results from large-scale unconditional cash transfer program in the United States, which consistently find increased spending on child-specific items, including books and toys, among families receiving monthly unconditional cash gifts [30,31]. Similarly, a study from Zambia reports a positive but small increase in the number of households with three or more children’s books following Child Support Grant Program [29]. The discrepancy from other existing studies may be explained by contextual differences. First, the local availability and affordability of children’s books could limit their purchase even when income increases. Second, it is possible that families prioritize more immediate needs such as food, housing, or medical care over educational investments when provided with flexible cash.

Our study also looks at how unconditional cash transfer influences children’s play environments in Thailand, particularly the types of toys children engage with. Our analysis reveals a significant increase in the proportion of children playing with homemade toys across both years while insignificant effect with regards to manufactured toys. This result is consistent with evidence from the US, where unconditional cash transfer is found to increase spending on child-specific goods including toys [30,31]. However, there is some slight difference from the experience of high-income country. The Thai case shows that even though 98% of children in the sample owns manufactured toys (see Table 1) there is a shift towards more cost-effective form of play namely homemade toys following the receipt of CSG. Thai households may reduce discretionary spending on children’s commercial toys due to inflation and economic uncertainty post-COVID 19 pandemic. Furthermore, increased homemade toys may also signal creative adaptation and resourcefulness on childrearing in developing country settings.

Finally, this research shows that CSG recipients are associated with the increase of more than 8 percentage point in electronic devices’ usage and on average 0.14 longer hours of electronic device usage per day than non-CSG recipients. To the best of our knowledge, this study is one of the first that looks at children outcomes in terms of electronic device access and duration of use. Our conjecture for the result is that grant receiver households with greater household resources can raise access to electronic devices such as TVs, computers, tablets, and smartphones, which can eventually lead to higher electronic device usage not only by adults but also by children living in those households. Furthermore, evidence from the US shows that parents spend more on child-related goods including child-related electronic devices once receiving cash transfer [30,31]. By analogy, families in Thailand might treat additional screen-based resources such as computer games as discretionary child enrichment activity and thus invest more in it.

### Strengths and limitations

This study has several strengths. First, it uses a large, nationally representative sample of children residing across all regions of Thailand. Second, the study evaluates a nationwide implementation of unconditional cash transfer program with substantial policy relevance in a middle-income country context where evidence remains limited. Third, we examine multiple child outcomes spanning developmental, educational materials, and technology domains, providing comprehensive assessment of CSG. Third, the use of propensity score matching improves the comparability of treatment and control groups by balancing observed characteristics and reducing selection bias, thereby strengthening the credibility of the estimated associations between CSG receipt and child development outcomes.

Several limitations should also be acknowledged. First, because the study is based on observational data, the findings should not be interpreted as definitive causal effects. Although propensity score matching accounts for observed confounding, the possibility of bias due to unobservable confounders cannot be ruled out. For example, parental motivation and parenting practice could be unmeasured confounders that may influence both grant participation and child outcomes. Second, the 2019 and 2022 MICS surveys employ different development assessment protocols when measuring whether a child is developmentally on track. Third, all child related outcomes in this study are self-reported by caretakers which may be subject to recall bias and measurement error. Fourth, we address missing data through complete-case analysis due to low level of missingness in our data. This approach may introduce selection bias if missingness is related to unmeasured factors. Lastly, the propensity score matching analysis in this study does not incorporate survey sampling weights and therefore may not fully account for the complex survey design of the Thai MICS survey. Consequently, the estimated treatment effects should be interpreted with some caution when generalizing the findings to the national population.

## Conclusion

This study provides empirical evidence on the association between Thailand’s unconditional CSG and early childhood outcomes using nationally representative data from the 2019 and 2022 MICS surveys. By employing propensity score matching approach, we attempt to estimate the effects of CSG receipt on several dimensions of children outcomes, including overall early childhood development, access to child-specific goods, and children’s interaction with electronic devices. Our findings suggest that the unconditional CSG has a modest but statistically significant positive association with children’s overall developmental status, supporting the notion that cash transfer can play a meaningful role in enhancing early childhood outcomes in a middle-income country context. This aligns with evidence from other LMICs, such as Ecuador, and complements earlier Thai-specific studies focused on nutritional outcomes. However, the results also reveal complex and sometimes unexpected patterns in how households allocate resources when receiving CSG. Notably, we observe a statistically significant decline in the number of children’s books in grant-receiving households, a finding that diverges from experiences in other countries and may reflect local constraints or differing household priorities. The grant appears to influence children’s play environments with an increase in engagement with homemade toys. The study also documents a substantial increase in children’s access to and time spent on electronic devices among grant-recipient households, which highlight both opportunities and potential risks related to screen exposure in early childhood. For policymakers, these findings emphasize the value of continued investment in cash transfer programs specifically for children while also suggesting the need for complementary intervention such as public campaigns on early childhood stimulation or parental guidance on electronic device’s use in order to maximize the benefits of such financial support. Future research could further explore the long-term consequences of increased screen time and the mechanisms behind resource allocation choices within households that receive unconditional child support grant.

## Supporting information

S1 Table Questions for assessing child development in 2019 and 2022. (DOCX)

S2 Table Sensitivity Analyses Full Result of Binary Logit Model (Average Marginal Effect). (DOCX)

S3 Table Sensitivity Analyses Full Result of OLS Regression (OLS coefficients). (DOCX)
