# Supplementary material for: Unconditional child support grant and outcomes on children: Experience from Thailand
Source: PLoS One. 2026 Aug 27;21(8):e0349423. doi: 10.1371/journal.pone.0349423 (PMC13521378; doi:10.1371/journal.pone.0349423)
Supplement: Supplementary file 1 [file pone.0349423.s001.docx]

| **S1 Table. Questions for Assessing Child Development in 2019 and 2022** | | |
| --- | --- | --- |
| **Questions from 2019 MICS** |  | **Questions from 2022 MICS** |
| **Literacy-Numeracy Domain** |  | Q1: Can a child walk on an uneven surface, for example a bumpy or steep road without falling? |
| Q1: Can a child identify or name at least ten letters of the alphabet? |  | Q2: Can a child jump up with both feet leaving the ground? |
| Q2: Can a child read at least four simple popular words? |  | Q3: Can a child dress him/herself, that is, put on pants and a shirt without help? |
| Q3: Does a child know the name and recognize the symbol of all numbers from 1 to 10? |  | Q4: Can a child fasten and unfasten buttons without help? |
| **Physical Domain** |  | Q5: Can a child say 10 or more words like "mama", "rice" or "water"? |
| Q4: Can a child pick up a small object with two fingers, like a stick or a rock from the ground? |  | Q6: Can a child speak using sentences of 3 or more words that go together, for example "I want water" or "The house is big"? |
| Q5: Is a child sometimes too sick to play? |  | Q7: Can a child speak using sentences of 5 or more words that go together, for example "The house is very big"? |
| **Approaches to Learning Domain** |  | Q8: Can a child correctly use any of the words "I", "you", "she" or "he", for example "I want water," or "He eats rice"? |
| Q6: Does a child follow simple directions on how to do something correctly? |  | Q9: If you show a child an object he/she knows well, such as a cup, bowl or animal, can he/she consistently name it? |
| Q7: When given something to do, is a child able to do it independently? |  | Q10: Can a child recognize at least 5 letters of the alphabet? |
| **Social-Emotional Domain** |  | Q11: Can a child write his/her own name? |
| Q8: Does a child get along well with other children? |  | Q12: an a child recognize all numbers from 1 to 5? |
| Q9: Does a child kick, bite, or hit other children or adults? |  | Q13: If you ask a child to give you 3 objects, such as 3 stones, 3 beans or 3 candies, does he/she give you the correct amount? |
| Q10: Does a child get distracted easily? |  | Q14: Can a child count 10 objects, for example 10 fingers or 10 blocks, without mistakes? |
|  |  | Q15: Can a child do an activity, such as colouring or playing with building blocks, without repeatedly asking for help or giving up too quickly? |
|  |  | Q16: Does a child ask about familiar people other than parents when they  are not there, for example "Where is Grandma?"? |
|  |  | Q17: Does a child offer to help someone who seems to need help? |
|  |  | Q18: Does a child get along well with other children? |
|  |  | Q19: How often does a child seem to be very sad or depressed? Would you say: daily, weekly, monthly, a few times a year or never? |
|  |  | Q20: Compared with children of the same age, how much does a child kick, bite, or hit other children or adults? Would you say: not at all, less, the same, more, or a lot more? |
|  |  |  |
|  |  | **Cutoff score by age for being developmentally on track in 2022** |
|  |  | Score 7 or above for age 24 - 29 months |
|  |  | Score 9 or above for age 30 - 35 months |
|  |  | Score 11 or above for age 36 - 41 months |
|  |  | Score 13 or above for age 42 - 47 months |
|  |  | Score 15 or above for age 48 - 59 months |
