# Supplementary material for: Unconditional child support grant and outcomes on children: Experience from Thailand
Source: PLoS One. 2026 Aug 27;21(8):e0349423. doi: 10.1371/journal.pone.0349423 (PMC13521378; doi:10.1371/journal.pone.0349423)
Supplement: Supplementary file 2 [file pone.0349423.s002.docx]

| **S2 Table. Sensitivity Analyses Full Result of Binary Logit Model (Average Marginal Effect)** | | | | | | |
| --- | --- | --- | --- | --- | --- | --- |
|  | *Child Development* | *Play Homemadetoy* | *Play Toyshop* | *Play Householdobject* | *Play Electronic* |  |
| *CSG Receipt* | 0.0170*** | 0.0241** | 0.00257 | -0.00742 | 0.0806*** |  |
|  | (0.00622) | (0.00937) | (0.00267) | (0.00647) | (0.00807) |  |
| *Child Age* | -0.0294*** | -0.00782 | 0.00241 | 0.00277 | 0.0639*** |  |
|  | (0.00386) | (0.00556) | (0.00159) | (0.00378) | (0.00478) |  |
| *Child Gender* | -0.0101* | 0.0146* | 0.000344 | -0.0314*** | 0.000846 |  |
|  | (0.00593) | (0.00882) | (0.00251) | (0.00611) | (0.00766) |  |
| *HeadHH Gender* | -0.00367 | 0.0129 | 0.00485* | 0.000358 | -0.000276 |  |
|  | (0.00603) | (0.00897) | (0.00253) | (0.00617) | (0.00780) |  |
| *HeadHH Age* | 0.000296 | -0.000759** | 0.000152 | 0.000229 | -0.000619** |  |
|  | (0.000227) | (0.000335) | (0.0000946) | (0.000232) | (0.000291) |  |
| *HH Number* | -0.00690*** | 0.0000647 | -0.00159*** | 0.00373* | 0.00525** |  |
|  | (0.00165) | (0.00264) | (0.000612) | (0.00192) | (0.00233) |  |
| *Caretaker Primary* | 0.0185 | 0.0106 | -0.00105 | 0.0204 | 0.0734*** |  |
|  | (0.0169) | (0.0277) | (0.00723) | (0.0194) | (0.0210) |  |
| *Caretaker Secondary* | 0.0229 | 0.0282 | -0.000334 | 0.0205 | 0.143*** |  |
|  | (0.0169) | (0.0276) | (0.00720) | (0.0193) | (0.0210) |  |
| *Caretaker Vocational* | 0.0408** | 0.0277 | -0.00208 | 0.0223 | 0.199*** |  |
|  | (0.0191) | (0.0301) | (0.00804) | (0.0210) | (0.0244) |  |
| *Caretaker University* | 0.0283 | 0.0421 | -0.00177 | 0.0209 | 0.154*** |  |
|  | (0.0186) | (0.0296) | (0.00801) | (0.0205) | (0.0233) |  |
| *Caretaker UniPlus* | 0.0323 | 0.0124 | N/A | 0.0244 | 0.203*** |  |
|  | (0.0309) | (0.0433) | N/A | (0.0281) | (0.0396) |  |
| *2nd Wealth Decile* | 0.0209* | -0.00229 | 0.00299 | -0.0576*** | 0.0795*** |  |
|  | (0.0119) | (0.0191) | (0.00440) | (0.0140) | (0.0150) |  |
| *3rd Wealth Decile* | 0.00759 | 0.00748 | 0.00426 | -0.0456*** | 0.0999*** |  |
|  | (0.0115) | (0.0189) | (0.00443) | (0.0141) | (0.0151) |  |
| *4th Wealth Decile* | 0.0153 | 0.0328* | 0.0157*** | -0.0321** | 0.108*** |  |
|  | (0.0117) | (0.0191) | (0.00530) | (0.0145) | (0.0152) |  |
| *5th Wealth Decile* | 0.0157 | 0.00467 | 0.0134*** | -0.0246* | 0.105*** |  |
|  | (0.0119) | (0.0191) | (0.00516) | (0.0148) | (0.0154) |  |
| *6th Wealth Decile* | 0.0458*** | 0.0412** | 0.0128** | -0.0222 | 0.134*** |  |
|  | (0.0128) | (0.0194) | (0.00508) | (0.0149) | (0.0158) |  |
| *7th Wealth Decile* | 0.0477*** | 0.0171 | 0.0234*** | -0.0561*** | 0.157*** |  |
|  | (0.0134) | (0.0199) | (0.00650) | (0.0146) | (0.0168) |  |
| *8th Wealth Decile* | 0.0400*** | 0.0402* | 0.0170*** | -0.0550*** | 0.176*** |  |
|  | (0.0137) | (0.0208) | (0.00590) | (0.0151) | (0.0181) |  |
| *9th Wealth Decile* | 0.0571*** | 0.0519** | 0.0174*** | -0.0654*** | 0.176*** |  |
|  | (0.0157) | (0.0227) | (0.00653) | (0.0159) | (0.0199) |  |
| *10th Wealth Decile* | 0.0638*** | 0.0393 | 0.0251*** | -0.0941*** | 0.144*** |  |
|  | (0.0177) | (0.0248) | (0.00860) | (0.0166) | (0.0217) |  |
| *Central* | -0.0125 | -0.0422* | -0.0158** | 0.0697*** | -0.00926 |  |
|  | (0.0168) | (0.0223) | (0.00786) | (0.0137) | (0.0202) |  |
| *North* | -0.00953 | 0.0775*** | 0.00455 | 0.0529*** | 0.0440** |  |
|  | (0.0173) | (0.0233) | (0.00857) | (0.0139) | (0.0210) |  |
| *Northeast* | -0.0351** | 0.0697*** | 0.00249 | 0.0253** | 0.0243 |  |
|  | (0.0164) | (0.0221) | (0.00804) | (0.0129) | (0.0199) |  |
| *South* | -0.00926 | 0.0548** | -0.00577 | 0.0659*** | 0.00404 |  |
|  | (0.0165) | (0.0219) | (0.00790) | (0.0131) | (0.0198) |  |
| *Urban* | 0.00603 | 0.0131 | 0.00245 | -0.0183*** | 0.0270*** |  |
|  | (0.00647) | (0.00961) | (0.00276) | (0.00655) | (0.00844) |  |
| N | 11464 | 11448 | 11238 | 11452 | 11453 |  |
| Notes: (1) Standard errors in brackets, * p<0.10, ** p<0.05, *** p<0.01, (2) 222 observations in the Play Toyshop model are dropped due to perfect prediction. | | | | | | |
