# Supplementary material for: Unconditional child support grant and outcomes on children: Experience from Thailand
Source: PLoS One. 2026 Aug 27;21(8):e0349423. doi: 10.1371/journal.pone.0349423 (PMC13521378; doi:10.1371/journal.pone.0349423)
Supplement: Supplementary file 3 [file pone.0349423.s003.docx]

| **S3 Table. Sensitivity Analyses Full Result of OLS Regression (OLS coefficients)** | | | | | | |  |
| --- | --- | --- | --- | --- | --- | --- | --- |
|  | *Child  Development* | *Children  Book* | *Play  Homemadetoy* | *Play  Toyshop* | *Play  Householdobject* | *Play  Electronic* | *Duration  Electronic* |
| *CSG Receipt* | 0.0174*** | -0.175*** | 0.0243*** | 0.00279 | -0.00713 | 0.0805*** | 0.202*** |
|  | (0.00631) | (0.0547) | (0.00941) | (0.00262) | (0.00645) | (0.00817) | (0.0284) |
| *Child Age* | -0.0283*** | 0.332*** | -0.00776 | 0.00227 | 0.00282 | 0.0634*** | 0.0991*** |
|  | (0.00372) | (0.0322) | (0.00555) | (0.00155) | (0.00380) | (0.00482) | (0.0166) |
| *Child Gender* | -0.0101* | -0.236*** | 0.0146* | 0.000346 | -0.0312*** | 0.000906 | 0.0475* |
|  | (0.00592) | (0.0513) | (0.00883) | (0.00246) | (0.00605) | (0.00767) | (0.0263) |
| *HeadHH Gender* | -0.00349 | 0.169*** | 0.0131 | 0.00505** | 0.000412 | 0.000462 | -0.0338 |
|  | (0.00604) | (0.0523) | (0.00900) | (0.00251) | (0.00617) | (0.00782) | (0.0268) |
| *HeadHH Age* | 0.000313 | 0.00189 | -0.000761** | 0.000169* | 0.000236 | -0.000620** | -0.000620 |
|  | (0.000225) | (0.00195) | (0.000336) | (0.0000937) | (0.000230) | (0.000292) | (0.00101) |
| *HH Number* | -0.00746*** | -0.106*** | 0.0000978 | -0.00207*** | 0.00348* | 0.00524** | 0.00477 |
|  | (0.00178) | (0.0154) | (0.00266) | (0.000740) | (0.00182) | (0.00231) | (0.00809) |
| *Caretaker Primary* | 0.0207 | 0.468*** | 0.0111 | -0.00129 | 0.0201 | 0.0997*** | -0.0754 |
|  | (0.0190) | (0.164) | (0.0283) | (0.00790) | (0.0194) | (0.0246) | (0.0971) |
| *Caretaker Secondary* | 0.0259 | 0.923*** | 0.0290 | -0.000354 | 0.0204 | 0.179*** | -0.0434 |
|  | (0.0189) | (0.164) | (0.0282) | (0.00786) | (0.0193) | (0.0245) | (0.0961) |
| *Caretaker Vocational* | 0.0420** | 1.252*** | 0.0284 | -0.00158 | 0.0223 | 0.222*** | -0.0407 |
|  | (0.0206) | (0.178) | (0.0307) | (0.00856) | (0.0210) | (0.0266) | (0.102) |
| *Caretaker University* | 0.0307 | 1.924*** | 0.0423 | -0.00159 | 0.0208 | 0.187*** | -0.166* |
|  | (0.0202) | (0.175) | (0.0301) | (0.00839) | (0.0206) | (0.0261) | (0.101) |
| *Caretaker UniPlus* | 0.0340 | 2.541*** | 0.0132 | 0.00938 | 0.0243 | 0.231*** | -0.198 |
|  | (0.0294) | (0.254) | (0.0438) | (0.0122) | (0.0300) | (0.0380) | (0.135) |
| *2nd Wealth Decile* | 0.0250* | 0.258** | -0.00240 | 0.00448 | -0.0536*** | 0.101*** | 0.135** |
|  | (0.0130) | (0.113) | (0.0194) | (0.00541) | (0.0133) | (0.0169) | (0.0622) |
| *3rd Wealth Decile* | 0.0102 | 0.468*** | 0.00767 | 0.00634 | -0.0405*** | 0.124*** | 0.0316 |
|  | (0.0129) | (0.111) | (0.0192) | (0.00534) | (0.0131) | (0.0167) | (0.0609) |
| *4th Wealth Decile* | 0.0194 | 0.697*** | 0.0331* | 0.0183*** | -0.0275** | 0.132*** | 0.0905 |
|  | (0.0129) | (0.111) | (0.0192) | (0.00534) | (0.0131) | (0.0167) | (0.0607) |
| *5th Wealth Decile* | 0.0202 | 0.908*** | 0.00480 | 0.0164*** | -0.0206 | 0.129*** | 0.182*** |
|  | (0.0130) | (0.113) | (0.0194) | (0.00540) | (0.0133) | (0.0168) | (0.0612) |
| *6th Wealth Decile* | 0.0488*** | 1.235*** | 0.0412** | 0.0159*** | -0.0189 | 0.158*** | 0.211*** |
|  | (0.0130) | (0.113) | (0.0195) | (0.00542) | (0.0133) | (0.0169) | (0.0609) |
| *7th Wealth Decile* | 0.0503*** | 1.627*** | 0.0175 | 0.0235*** | -0.0510*** | 0.178*** | 0.200*** |
|  | (0.0135) | (0.117) | (0.0201) | (0.00560) | (0.0138) | (0.0174) | (0.0623) |
| *8th Wealth Decile* | 0.0442*** | 2.053*** | 0.0404* | 0.0198*** | -0.0499*** | 0.192*** | 0.164** |
|  | (0.0140) | (0.121) | (0.0209) | (0.00581) | (0.0143) | (0.0181) | (0.0640) |
| *9th Wealth Decile* | 0.0579*** | 2.741*** | 0.0518** | 0.0201*** | -0.0615*** | 0.193*** | 0.203*** |
|  | (0.0151) | (0.131) | (0.0226) | (0.00629) | (0.0155) | (0.0196) | (0.0685) |
| *10th Wealth Decile* | 0.0633*** | 3.738*** | 0.0398 | 0.0248*** | -0.0989*** | 0.166*** | 0.187** |
|  | (0.0166) | (0.144) | (0.0248) | (0.00691) | (0.0170) | (0.0215) | (0.0748) |

| **S3 Table. Sensitivity Analyses Full Result of OLS Regression (OLS coefficients)** | | | | | | |  |
| --- | --- | --- | --- | --- | --- | --- | --- |
| **(continued)** |  |  |  |  |  |  |  |
|  | *Child  Development* | *Children  Book* | *Play  Homemadetoy* | *Play  Toyshop* | *Play  Householdobject* | *Play  Electronic* | *Duration  Electronic* |
| *Central* | -0.0102 | -1.081*** | -0.0467** | -0.0175*** | 0.0863*** | -0.0114 | -0.171** |
|  | (0.0153) | (0.133) | (0.0229) | (0.00637) | (0.0157) | (0.0198) | (0.0670) |
| *North* | -0.00782 | -0.994*** | 0.0773*** | 0.00428 | 0.0708*** | 0.0407** | -0.238*** |
|  | (0.0158) | (0.137) | (0.0235) | (0.00655) | (0.0161) | (0.0204) | (0.0687) |
| *Northeast* | -0.0343** | -1.618*** | 0.0700*** | 0.00391 | 0.0424*** | 0.0219 | -0.292*** |
|  | (0.0150) | (0.130) | (0.0224) | (0.00624) | (0.0153) | (0.0194) | (0.0657) |
| *South* | -0.00789 | -1.683*** | 0.0552** | -0.00394 | 0.0826*** | 0.00268 | -0.371*** |
|  | (0.0149) | (0.130) | (0.0223) | (0.00620) | (0.0153) | (0.0193) | (0.0652) |
| *Urban* | 0.00598 | 0.229*** | 0.0130 | 0.00258 | -0.0179*** | 0.0266*** | -0.0253 |
|  | (0.00643) | (0.0557) | (0.00958) | (0.00267) | (0.00657) | (0.00832) | (0.0283) |
| *_cons* | 0.963*** | 1.221*** | 0.601*** | 0.958*** | 0.821*** | 0.177*** | 0.877*** |
|  | (0.0306) | (0.265) | (0.0456) | (0.0127) | (0.0312) | (0.0396) | (0.145) |
| N | 11464 | 11457 | 11448 | 11460 | 11452 | 11453 | 8730 |
| Note: Standard errors in brackets, * p<0.10, ** p<0.05, *** p<0.01 | | | |  |  |  |  |
